# Prevalence of symptoms in children with acute lymphoblastic leukaemia: a systematic review and meta-analysis

**DOI:** 10.1186/s12885-023-11581-z

**Published:** 2023-11-15

**Authors:** Xiaoyan Lan, Junjun Wu, Zhenling Liao, Yong Wu, Rong Hu

**Affiliations:** 1 School of Nursing, Fujian Medical University, No. 1 Xueyuan Road, Shangjie Town, Minhou County, Fuzhou, 350108 Fujian Province China; 2 Department of Haematology, Fujian Medical University Union Hospital, 29 Xinquan Road, Fuzhou, 350001 China

**Keywords:** Acute lymphoblastic leukaemia, Signs and symptoms, Prevalence, Systematic review

## Abstract

**Background:**

Children with acute lymphoblastic leukaemia (ALL) experience multiple symptoms that occur in complicated patterns and negatively affect patient outcomes. To date, no systematic review has been performed on the prevalence of symptoms in children with ALL.

**Objective:**

The study aimed to report and analyse the prevalence of symptoms in children with ALL during treatment.

**Methods:**

A systematic search was conducted in eight databases (PubMed, Ovid Embase, Web of Science, CINAHL, PsycINFO, China WanFang Database, China Science and Technology Journal Database, and China National Knowledge Infrastructure) for studies published between January 1, 2000, and August 12, 2023. The methodological quality of the included studies was evaluated and a meta-analysis was performed to pool the prevalence of symptoms.

**Results:**

In total, 17 studies were included, from which 34 symptoms were identified. The symptom prevalence ranged between 1.5 and 91.0% and the most frequent symptoms observed were fatigue, lack of energy, dry mouth, lack of appetite, sweating, and feeling irritable, which occurred in at least 60% of the patients.

**Conclusions:**

Symptoms remain highly prevalent in paediatric patients with ALL, which provides support for the need for symptom assessment in the clinical setting. Specific intervention is urgently needed to mitigate the symptoms in children with ALL and help them cope with the symptom burden.

**Supplementary Information:**

The online version contains supplementary material available at 10.1186/s12885-023-11581-z.

## Introduction

Acute lymphoblastic leukaemia (ALL) is the most common malignancy in children worldwide [1]. The overall age-standardised incidence rate of leukaemia is 48.4 per million person-years in children aged 0–14 years [2]. Due to the improvements in the treatment of paediatric ALL over the past several decades, the 5-year survival rate now exceeds 90% in most developed countries [3].

Previous studies have suggested that paediatric patients with ALL often experience various symptoms, such as lack of energy, sweating, lack of appetite, nausea, and vomiting [4, 5], which in turn affected the patient’s outcome and the quality of life (QoL) [6]. To optimise the QOL in children with ALL, a comprehensive symptom assessment is needed to achieve symptom control. However, evidence-based criteria on how often symptoms should be assessed and which symptoms should be prioritised for assessment among children with ALL remains unknown [7].

Several reviews on the multiple symptoms experienced by children with cancer have been published [8, 9]. Although inferences can be made from these studies, paediatric ALL might present a different set of symptoms. Indeed, the findings of the Children’s Oncology Group State of the Science Symposium on Symptom Distress (2018) suggested that the most commonly reported symptoms were inconsistent among different cancer types [10]. This suggests that identifying the symptom profile for specific diseases and treatment groups is important for developing targeted interventions and preventive guidance to minimise symptom-related distress. To date, no systematic review has been performed on the prevalence of symptoms in children with ALL. Therefore, a systematic review and meta-analysis were performed to identify and analyse the prevalence of the symptoms in children with ALL that has been reported in clinical settings.

## Methods

### Protocol and registration

This systematic review and meta-analysis was performed in accordance with the Preferred Reporting Items for Systematic Reviews and Meta-analysis guidelines [11] and registered on PROSPERO (CRD42021269421) (https://www.crd.york.ac.uk/PROSPERO/).

### Search strategies and data sources

A systematic electronic search was conducted across eight databases (PubMed, Ovid Embase, Web of Science, CINAHL, PsycINFO, China WanFang Database, China Science and Technology Journal Database, and China National Knowledge Infrastructure) between January 1, 2000, and August 12, 2023. The bibliographies of relevant reviews and articles were hand-searched for potential studies for inclusion. The search terms were developed using free and subject terms and were combined with the Boolean operator OR/AND. The keywords included ‘child’, ‘paediatric’, ‘leukaemia’, and ‘symptom’. The search was restricted to peer-reviewed journal articles published in English and Chinese. Appropriate methodological filters were used for specific databases, where applicable. Other types of grey literature (e.g., conference abstracts) were excluded owing to the lack of details on the study methodology or findings. All retrieval strategies are shown in Supplemental Table S1.

### Eligibility criteria

The following inclusion criteria were used to select full-text articles: (1) quantitative design; (2) reporting on the prevalence of symptoms in children aged ≤ 18 years diagnosed with ALL; and (3) published in English or Chinese peer-reviewed journals. The minimum sample size was limited to 30 in observational studies to avoid selection bias from small studies. Studies with heterogeneous populations of patients with cancer were also included if the results for the patients with ALL were analysed separately. Studies with participants aged > 18 years were included if a separate analysis was performed for patients aged ≤ 18 years.

The exclusion criteria were: (1) the study design did not report empirical data (e.g. opinions, case reports, reviews, or editorials); (2) studies that focused on children receiving palliative care or consisting childhood cancer long-term survivors; (3) measuring symptoms using single symptom items drawn from the QoL or health status measures (e.g. Paediatric Quality of Life Inventory-4.0 Generic Core or Paediatric Quality of Life Inventory-Cancer Module; studies using such scoring systems were excluded because they focused on health-related QoL); and (4) measuring symptoms using an unvalidated scale.

### Study selection

Citations were imported to EndNote X9 (www.myendnoteweb.com/), and duplicates were removed. The study selection process was carried out by two investigators. After duplicate studies were excluded, the investigators independently assessed records based on the titles, abstracts, and full texts. If there was any disagreement between researchers, a third researcher settled the issue.

### Data extraction and synthesis

The data were collected from the included studies by two independent researchers using a standardised data sheet comprising the following items: authors, country of the study, publication year, study design, sample characteristics, and main findings. Any disagreement was resolved by a third author. The mean prevalence was computed by averaging the reported values across the available time points in longitudinal studies. When studies used different terminology to describe the same symptoms, the terms used by the original authors were retained to preserve the intended meaning. We synthesised the extracted data and presented the findings as narrative descriptions and descriptive statistics.

### Quality appraisal

The data quality was critically appraised using the Joanna Briggs Institute (JBI) Meta-Analysis tool for cross-sectional,and case-control studies [12]. The revised JBI tool for cohort studies was used for longitudinal studies because items 1, 2, and 6 were not applicable. Two investigators independently performed bias assessments. Any discrepancies in judgement regarding the risk of bias were resolved by discussion with a third review author acting as an arbiter, if necessary.

### Data analysis

We conducted meta-analysis when at least 2 studies reported comparable data measuring the same outcomes. The pooled frequency of symptoms was computed with weighted mean and standard errors, using a 95% confidence interval (CI) [13]. Heterogeneity among the studies was assessed using Cochran’s Q statistic, and heterogeneity was considered to be present when *P* < .05. The magnitude of heterogeneity was measured using *I*-square (*I* ^2^) statistic. *I* ^2^ values of 25%, 50%, and 75% were considered to indicate low, moderate, and high heterogeneity, respectively [14]. Random-effects model results were presented for data with high heterogeneity. Otherwise, fixed-effect model results were reported. A two-sided *P* < .05 indicated statistical significance. Other analyses (i.e., subgroup analysis, sensitivity analyses, and publication bias) were not performed because the included studies were insufficient for analysis. All statistical analyses were performed using Comprehensive Meta-Analysis version 3 (http//www.meta-analysis.com).

## Results

### Search results

**Fig. 1:**
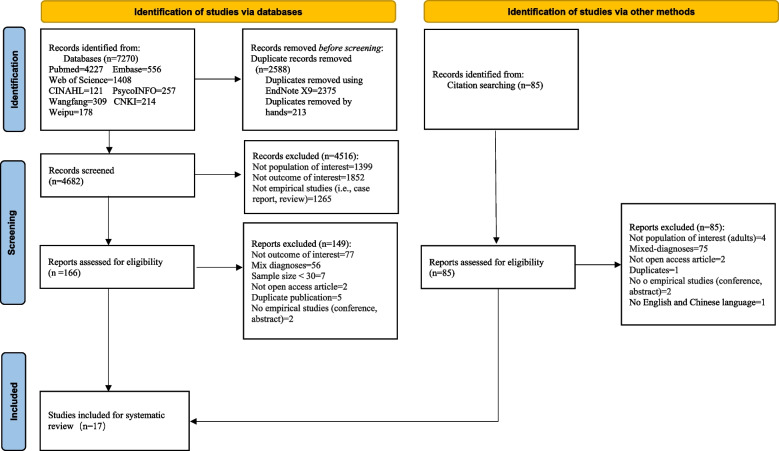
PRISMA 2020 flow diagram

The search yielded 7270 studies, of which 2588 were duplicates. After screening the titles and abstracts, 166 studies were included for full-text evaluation, and 17, [15–31] met the inclusion criteria. Furthermore, 85 studies were identified via a reference search but none met the inclusion criteria. Details of the screening process are shown in the Fig. 1.

### Characteristics of studies

In total, 17 studies published between 2010 and 2023 were included, and their characteristics are summarised in Table 1. Of the 17 included studies, 5 were longitudinal studies, and 12 were cross-sectional studies. There were a total of 1719 participants from seven countries: China (*n* = 6), Canada (*n* = 4), the United States (*n* = 3), the Netherlands (*n* = 2), Indonesia (*n* = 1), Australia (*n* = 1), and Dutch (*n* = 1). The sample size ranged between 34 and 216, and most of the included studies (*n* = 9, 52.94%) recruited children with a wide age range (2–18 years), but three studies enrolled children aged < 9 years [15–17].

**Table 1:** Summary of the included studies

Author, Year, and Country	Title	Design	Year of Data Retrieval	Sample Characteristics	Symptom Characteristics	Symptom(s) and Prevalence
Zupanec et al. 2010 [18] Canada	Sleep habits and fatigue of children receiving maintenance chemotherapy for ALL and their parents	Mixed methods – Cross-sectional design	2008.09-2009.01	*N* = 64; 51 (79.69%) male Age: 4–18 y (Mean age was not reported) Diagnoses: ALL Treatment state: Had finished the first 3 courses of maintenance chemotherapy Risk state: Standard risk (60.94%) High risk (29.69%) T-cell (9.38%) Race: Caucasian (51.56%) Asian (25.00%) Multiracial (14.06%) Black (7.81%) Hispanic (1.56%)	Symptom focus: Fatigue: FSA, FSP Sleep disturbance: CSHQ Reporting mode: < 13 y: Parent proxy report ≧ 13 y: Children self-report Recall period: 1 week	Sleep disturbance (85.94%)
Bu et al. 2015 [28] China	Study of fatigue and related factors among children with acute lymphoblastic leukemia receiving chemotherapy in hospital (Chinese version)	Quantitative -Cross-sectional design	2013.07-2014.01	*N* = 100; 65 (65.0%) male Age: 2–15 y (6) Diagnoses: ALL Treatment state: Receiving chemotherapy Risk state and race were not reported	Symptom focus: Fatigue: PedsQL^TM^MFS (Parent) Pain: Wong-Baker Faces Scale Reporting mode: Parents proxy-report Recall period: The recall period was not reported	Sleep disturbance (6.15%)
Ren et al. 2017 [19] China	Fatigue of children with acute lymphoblastic leukemia during chemotherapy and influencing factors (Chinese version)	Quantitative -Cross-sectional design	2014.02-2016.08	*N* = 216; 102 (47.2%) male Age: 5–15 y (Mean age was not reported) Diagnoses: ALL Treatment state: Receiving chemotherapy Risk state and race were not reported	Symptom focus: Fatigue: PedsQL^TM^MFS Other symptoms: MSAS10-18 Reporting mode: 5-8y: Parents proxy-report > 8: Children self-report Recall period: 1 week	Fatigue (95.83%)
Daniel et al. 2018 [20] USA	The relationship between child and caregiver sleep in acute lymphoblastic leukemia maintenance	Quantitative -Cross-sectional design	2010.05-2014.10	*N* = 68 (The gender information was not reported) Age: 3–12 y (Mean age was not reported) Diagnoses: ALL Treatment state: Maintenance phase of chemotherapy Risk state: Low (2.9%) Standard (63.2%) High (33.8%) Race: Caucasian (76.5%) Asian (4.4%) Black or African American (7.4%) Other (2.7%) More than one race (8.8%) Hispanic/Latino (10.3%)	Symptom focus: Sleep disturbance: CSHQ Reporting mode: Parents proxy report Recall period: 1 week	Sleep disturbance (67.65%)
Fadhilah et al. 2019 [21] Indonesia	The Relationship between Activity Level and Fatigue in Indonesian Children with Acute Lymphocytic Leukemia in the Home Setting	Quantitative -Cross-sectional design	The year of data retrieval was not reported	*N* = 45; 30 (66.7%) male Age: 3–16 y (Mean age was not reported) Diagnoses: ALL Treatment state: Receiving chemotherapy Induction (13.3%) Consolidation (6.7%) Maintenance (53.3%) Remission (26.7%) Risk state: Standard risk (62.2%) High risk (37.8%) The race was not reported	Symptom focus: Fatigue: Allen-Child Oncology Fatigue questionnaire Reporting mode: Primary self-report, partially report with the help of parents Recall period: The recall period was not reported	Fatigue (100%)
Loves et al. 2019 [22] Canada	Taste changes in children with cancer and hematopoietic stem cell transplant recipients	Quantitative -Cross-sectional design	2014.09-2017.06	*N* = 64 (The gender information was not reported) Age: The tumour type of sample was mixed, and the mean age of children with ALL was not reported, but all the participants were between 8–18 years old (Mean age was not reported) Diagnoses: ALL Treatment state: Receiving chemotherapy The risk state and race were not reported	Symptom focus: Taste changes: SSPedi Reporting mode: Children self-report Recall period: The recall period was not reported	Taste changes (40.6%)
Tomlinson et al. 2019 [23] Canada	Severely bothersome fatigue in children and adolescents with cancer and hematopoietic stem cell transplant recipients	Quantitative -Cross-sectional design	2014.09-2017.06	*N* = 64 (The gender information was not reported) Age: The tumour type of sample was mixed, and the mean age of children with ALL was not reported, but all the participants were between 8–18 years old (Mean age was not reported) Diagnoses: ALL Treatment state: Receiving chemotherapy The risk state and race were not reported	Symptom focus: Fatigue: SSPedi Reporting mode: Children self-report Recall period: The recall period was not reported	Fatigue (14.1%)
Ma et al. 2019 [30] China	Analysis of the correlation between fatigue and discomfort symptoms in children with acute lymphoblastic Leukemia and its influencing factors (Chinese version)	Quantitative -Cross-sectional Design	2017.01-2018.03	*N* = 68; 27 (39.7%) male Age: 5–15 y (8.29) Diagnoses: ALL Treatment state: Receiving chemotherapy The risk state and race were not reported	Symptom focus: Fatigue: PedsQL^TM^MFS Other symptoms: MSAS 10–18 Reporting mode: Primary self-report, partially reported with the help of parents Recall period: 1 week	Fatigue (95.59%)
Hyslop et al. 2021 [24] Canada	Feeling scared or worried self-report in children receiving cancer treatments using the Symptom Screening in Pediatrics Tool (SSPedi)	Quantitative -Cross-sectional design	2014.09-2017.07	*N* = 64 (The gender information was not reported) Age: The tumour type of sample was mixed, and the mean age of children with ALL was not reported, but all the participants were between 8–18 years old (Mean age was not reported) Diagnoses: ALL Treatment state: Receiving chemotherapy The risk state and race were not reported	Symptom focus: Feeling scared or worried: SSPedi Reporting mode: Children self-report Recall period: The recall period was not reported	Feeling scared or worried (40.77%)
Zhou et al. 2021 [25] China	Cancer-related fatigue status and influencing factors in children with acute lymphoblastic leukemia (Chinese version)	Quantitative -Cross-sectional design	2018.12-2019.09	*N* = 102; 62 (60.78%) male Age: 5–18 y (Mean age was not reported) Diagnoses: ALL Treatment state: Induction (41.18%) Consolidation (44.12%) Maintenance (14.70%) Risk state: low risk (34.31%) Moderate risk (41.18%) High risk (24.51%) The race was not reported	Symptom focus: Fatigue: PedsQL^TM^MFS Reporting mode: Children self-report Recall period: 1 month	Fatigue (98.04%)
Hockenberry et al. [29] 2014 USA	The influence of oxidative stress on symptom occurrence, severity, and distress during childhood Leukemia treatment	Quantitative - Prospective Longitudinal Design	The year of data retrieval was not reported	*N* = 34; 17 (47.2%) male Age: 3–15 y (7.36) Diagnoses: ALL Treatment state: Receiving chemotherapy The risk state was not reported Race: Caucasian (44.4%) Hispanic (41.7%) African American (5.6%) Native American (2.8%) Other (5.6%)	Symptom focus: Multiple symptoms: MSAS10-18 Reporting mode: 3–7: Parents proxy report 8-15: Children self-report Data collection points: T1-T6: Average of 45, 142, 241, 338, 424, and 510 days from diagnosis, respectively, spanning induction, post-induction, and during continuation therapy Recall period: 1 week	Lack of energy (47.06%) Pain (41.18%) Feeling drowsy (29.41%) Nausea (41.18%) Cough (41.18%) Lack of appetite (44.12%) Feeling sad (29.41%) Feeling nervous (35.29%) Worrying (20.59%) Feeling irritable (47.06%) Itching (23.53%) Insomnia (29.41%) Hair loss (50.00%) Vomiting (23.53%) Weight loss (26.47%) Sweating (29.41%) Lack of concentration (38.24%) Diarrhea (14.71%) Skin changes (20.59%) Dyspnea (14.71%) Change in the way food tastes (41.18%) “I don’t look like myself” (17.65%) Mouth sores(20.59%) Constipation (17.65%)
Kunin-Batson et al. 2016 [17] USA	Prevalence and predictors of anxiety and depression after completion of chemotherapy for childhood acute lymphoblastic leukemia: A prospective longitudinal study	Quantitative - Prospective Longitudinal Design	2005–2009	*N* = 159; 83 (52.2%) male Age: 2–9 y (Mean age was not reported) Diagnoses: ALL Treatment state: Receiving chemotherapy The risk state was not reported Race: White, non-Hispanic (67.9%) Black, non-Hispanic (6.9%) Hispanic (16.4%) Other (8.8%)	Symptom focus: Anxiety and depression: BASC-2 Reporting mode: Parents proxy report Data collection points: T1: day 1 of consolidation therapy T2: the end of the delayed intensification T3: 6 months after the initiation of maintenance therapy T4: 3 months after the completion of therapy Recall period: 1 month	Anxiety (24.8%) Depression (27.6%)
McCarthy et al. 2016 [16] Australia	Are parenting behaviors associated with child sleep problems during treatment for acute lymphoblastic leukemia?	Quantitative- Cross-sectional & Case-control design	The year of data retrieval was not reported	*N* = 43; 30 (69.77%) male Age: 2–6 y (4.6) Diagnoses: ALL Treatment state: During the maintenance phase of ALL treatment The risk state and race were not reported	Symptom focus: Sleep disturbance: TCSQ Reporting mode: Parents proxy-report Recall period: The recall period was not reported	Sleep disturbance (48.00%)
Li et al. 2019 [31] China	Symptom clusters among children with acute lymphocytic leukemia during chemotherapy: a longitudinal study(Chinese version)	Quantitative - Prospective Longitudinal Design	2017.07-2018.11	*N* = 130; 85 (63.4%) male Age: 8–16 y (10.53) Diagnoses: ALL Treatment state: Receiving chemotherapy Risk state: Low risk (20.15%) Moderate risk (45.93%) High risk (33.58%) The race was not reported	Symptom focus: Multiple symptoms: MSAS10-18 Reporting mode: Children self-report Data collection points: Four data collection points: T1: Before chemotherapy T2: induction T3: consolidation T4: maintenance of chemotherapy Recall period: 1 week	Lack of energy (94.62%) Pain (68.46%) Headache (36.92%) Feeling drowsy (46.92%) Nausea (70.00%) Cough (53.08%) Lack of appetite (83.85%) Feeling sad (53.85%) Feeling nervous (66.15%) Worrying (64.62%) Feeling irritable (70.00%) Itching (23.85%) Insomnia (60.77%) Dry mouth (68.46%) Hair loss (53.85%) Vomiting (63.85%) Weight loss (67.69%) Dizziness (41.54%) Numbness/tingling in hands/feet (18.46%) Sweating (85.38%) Lack of concentration (51.54%) Diarrhea (7.69%) Skin changes (28.46%) Dyspnea (2.31%) Change in the way food tastes (83.85%) “I don’t look like myself” (53.85%) Mouth sores (24.62%) Constipation (60.77%) Swelling of arms/legs (5.38%) Problems with urination (1.54%)
Steur et al. 2020 [15] Netherlands	High prevalence of parent-reported sleep problems in pediatric patients with acute lymphoblastic leukemia after induction therapy	Quantitative - Prospective Longitudinal Design	The year of data retrieval was not reported	*N* = 113; 63 (55.8%) male Age: 3–9 y (4.8) Diagnoses: ALL Treatment state: Receiving chemotherapy Risk state: Standard (26.55%) Medium (73.45%) The race was not reported	Symptom focus: Fatigue: PedsQL MFS-parent Sleep: Actigraphy, sleep diary Reporting mode: Parents proxy-report Data collection points: T1: After induction, T2: Between two hospital admissions Recall period: 1 week	Sleep disturbance (12.10%)
Irestorm et al., 2023 [27] Dutch	Fatigue trajectories during pediatric ALL therapy are associated with fatigue after treatment: a national longitudinal cohort study	Quantitative - Prospective Longitudinal Design	The year of data retrieval was not reported	*N* = 92 Age: 2–18 y Diagnoses: ALL Treatment state: Receiving chemotherapy Risk state: Standard (25%) Medium (75%) The race was not reported	Symptom focus: Fatigue: PedsQL MFS-parent Reporting mode: Parents proxy report Data collection points: T0:5 months after diagnosis, T1: 12 months after diagnosis T2: 24 months after diagnosis Recall period: 1 week	Fatigue (78.26%)
Xi et al., 2023 China [26]	Analyzing sleep status in children with acute leukemia	Quantitative- Cross-sectional design	2020 to 2022	*N* = 173; 96 (55.5%) male Age: 0–18 y (6.86) Diagnoses: ALL(*n* = 167) Treatment state: Receiving chemotherapy The risk state was not reported The race was not reported	Symptom focus: Sleep disturbance: CSDS Reporting mode: 0–7: Parents proxy report 8-18: Children self-report Recall period: Not report	Sleep disturbance (40.12%)

*Abbreviations*: *BASC-2 *The Behavioral Assessment System for Children-2nd Edition: Parent Report Scale, *CSHQ *Children's Sleep Habits Questionnaire, *FSA *Fatigue Scale–Adolescent, *FSP *Fatigue Scale–Parent; PedsQL^TM^ *MFS *Multidimensional Fatigue Scale, *MSAS10-18 *Memorial Symptom Assessment Scale 10-18, *SSPedi *Symptom Screening in Pediatrics Tool, *TCSQ *Tayside children’s sleep questionnaire

Of the included studies, eight focused on multiple symptoms and nine on a single symptom. Of the nine studies that focussed on a single symptom, four discussed fatigue [21, 23, 25, 27], followed by sleep disturbance (*n* = 3) [16, 20, 26], taste alteration (*n* = 1) [22], and feeling scared or worried (*n* = 1) [24]. The tools to measure these symptoms, including those measuring single symptoms, and multi-symptom inventories, are listed in Table 2. Fatigue is most often assessed by the PedsQL^TM^MFS (*n* = 6). The MSAS 10–18 was the most commonly used scale to measure multiple symptoms (*n* = 5).

**Table 2:** Instrument List (*N* = 10)

Instruments	Symptoms
Multidimensional Fatigue Scale (PedsQL™ MFS)	Fatigue
Fatigue Scale–Adolescent (FSA)	Fatigue
Fatigue Scale–Parent (FSP)	Fatigue
Allen-Child Oncology Fatigue questionnaire	Fatigue
Children’s Sleep Habits Questionnaire (CSHQ)	Sleep Disturbance
Tayside Children’s Sleep Questionnaire (TCSQ)	Sleep Disturbance
Wong-Baker Faces Scale	Pain
The Behavioral Assessment System for Children-2nd Edition (BASC-2)	Anxiety and depression
Memorial Symptom Assessment Scale 10–18 (MSAS10-18)	Multiple symptoms
Symptom Screening in Pediatrics Tool (SSPedi)	Multiple symptoms

Three categories of symptom reporters existed: only children reporters (*n* = 5), children and parent reporters (*n* = 6), and only parent reporters (*n* = 6). In longitudinal studies, the length of follow-up time ranged from 7 days to 18 months. One study included 3 months follow-up after treatment completion [17]. Of the 17 studies, most explored symptom experiences during ALL therapy without distinguishing between treatment stages. Three studies focused on the maintenance chemotherapy period [16, 18, 20].

### Methodological quality of included studies

The details of the evaluation process are presented in Supplemental Tables S2, S3 and S4. The lack of identification of confounding factors and strategies to deal with confounding factors (*n* = 5) and lack of strategies to address incomplete follow-up (*n* = 3) were the main reasons for the risk of bias.

### Symptom prevalence

**Fig. 2:**
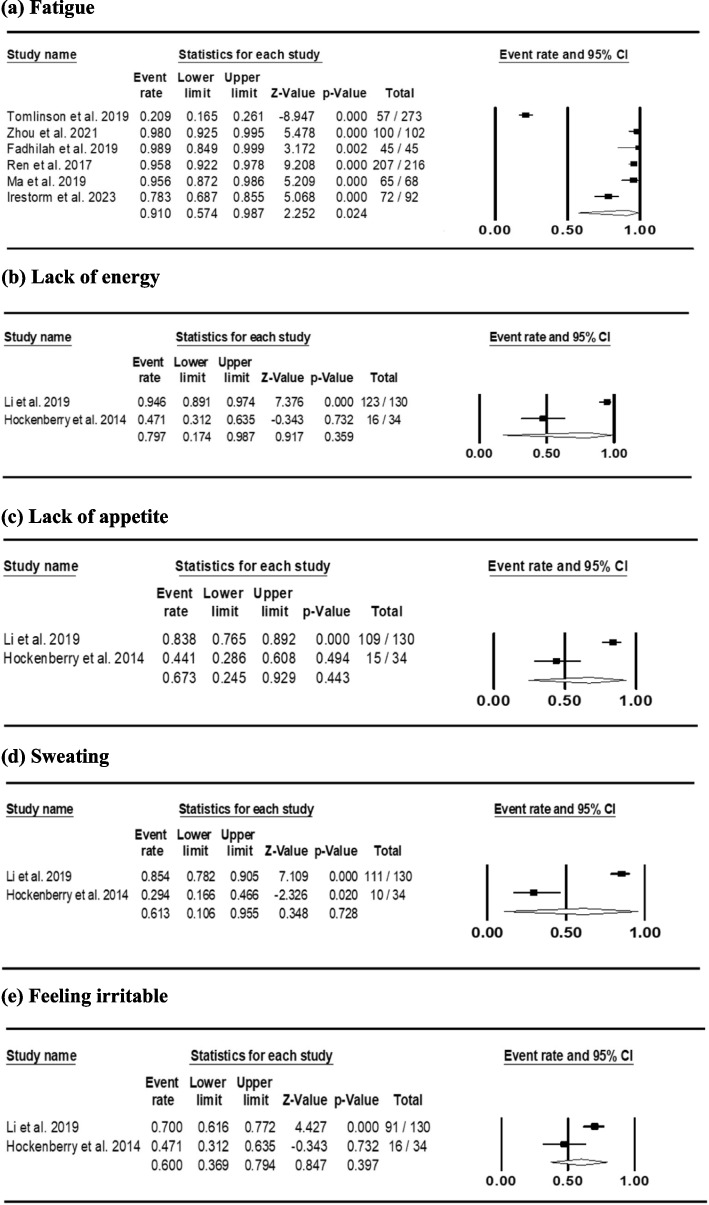
Forest plot of the most common symptoms in childhood acute lymphoblastic leukaemia with a pooled prevalence estimate of ≥ 60%

Thirty-four symptoms were identified across the seventeen studies. The symptom frequency ranged from 1.5% (urinary problems) to 91.0% (fatigue). A meta-analysis was performed (if available) to combine the symptom data from multiple studies (Table 3). Among the physical symptoms, the pooled prevalence for fatigue (6 studies; 796 patients) was 91.0% (95% CI 57.4–98.7%). The pooled prevalence for lack of energy (2 studies; 164 patients) was 79.7% (95% CI 17.4–98.7%). The pooled prevalence for dry mouth (1 studies; 89 patients) was 68.5% (95% CI 60.0–75.9%). The pooled prevalence for lack of appetite (2 studies; 164 patients) was 67.3% (95% CI 24.5–92.9%). And the pooled prevalence for sweating (2 studies; 164 patients) was 61.3% (95% CI 10.6–95.5%). Among the psychological symptoms, the pooled prevalence for feeling irritable (2 studies; 164 patients) was 60.0% (95% CI 36.9–79.4%). The pooled prevalence for feeling nervous (2 studies; 164 patients) was 51.7% (95% CI 23.5–78.9%). The pooled prevalence for worrying (3 studies; 451 patients) was 42.5% (95% CI 23.6–63.9%). And the pooled prevalence for feeling sad (2 studies; 164 patients) was 42.4% (95% CI 21.2–66.7%). Forest plots for the most common symptoms with a pooled prevalence estimate of more than 60% are shown in Fig. 2.

**Table 3:** Meta-analyses of symptom frequency (Pooled Estimates and Heterogeneity of Included Articles per Symptom)

Symptoms	*k*	*n*	Prevalence (%)	Heterogeneity				
				95% CI	Q	df(Q)	*I*,%	P
Fatigue	6	796	91.0^a^	57.4–98.7	257.28	5	98.06%	0.000
Lack of energy	2	164	79.7^a^	17.4–98.7	33.097	1	96.98%	0.000
Dry mouth	1	89	68.5^c^	60.0-75.9				
Lack of appetite	2	164	67.3^a^	24.5–92.9	20.14	1	95.04%	0.000
Sweating	2	164	61.3^a^	10.6–95.5	34.296	1	97.08%	0.000
Feeling irritable	2	164	60.0^a^	36.9–79.4	6.02	1	83.39%	0.014
Change in the way food tastes	3	437	57.7^a^	26.0-84.1	57.45	2	96.52%	0.000
Nausea	2	164	57^a^	29.0-81.1	9.171	1	89.10%	0.002
Pain	2	164	56.1^a^	29.7–79.5	8.155	1	87.74%	0.004
Hair loss	2	164	53^a^	45.4–60.6	0.16	1	0	0.689
Feeling nervous	2	164	51.7^a^	23.5–78.9	9.99	1	89.99%	0.002
Cough	2	164	50.6^b^	43.0-58.3	1.512	1	33.87%	0.219
Lack of concentration	2	164	48.9^b^	41.2–56.5	1.89	1	46.95%	0.17
Weight loss	2	164	47.3^a^	13.8–83.4	16.65	1	94.00%	0.000
Insomnia	2	164	45.6^a^	18.8–75.2	9.91	1	89.91%	0.002
Vomiting	2	164	43.3^a^	12.1–80.9	15.52	1	93.56%	0.000
Worrying	3	451	42.5^a^	23.6–63.9	28.29	2	92.93%	0.000
Feeling sad	2	164	42.4^a^	21.2–66.7	6.141	1	83.72%	0.013
Dizziness	1	130	41.5^c^	33.4–50.2				
Sleep disturbance	6	531	39.6^a^	18.1–65.1	110.53	5	95.48%	0.000
Feeling drowsy	2	164	39.5^a^	24.1–57.4	3.279	1	69.5·%	0.07
Constipation	2	164	37.6^a^	8.0-80.7	16.68	1	94.00%	0.000
Headache	1	130	36.9^c^	29.1–45.5				
“I don’t look like myself”	2	164	34.5^a^	9.1–73.4	12.31	1	91.88%	0.000
Depression	1	159	27.6^c^	19.9–36.9				
Skin changes	2	164	27^b^	20.7–34.3	0.84	1	0	0.359
Anxiety	1	159	24.8^c^	17.4–33.9				
Itching	2	164	23.8^b^	17.9–30.9	0.001	1	0	0.969
Mouth sores	2	164	23.8^b^	17.9–30.9	0.24	1	0	0.624
Numbness/tingling in hands/feet	1	130	18.5^c^	12.7–26.1				
Diarrhoea	2	164	9.5^b^	5.8–15.2	1.54	1	35.15%	0.214
Dyspnoea	2	164	6.2^a^	0.9–31.5	6.86	1	85.43%	0.009
Swelling of arms/legs	1	130	5.4^c^	2.6–10.9				
Problems with urination	1	130	1.5^c^	0.4–5.9				

*Abbreviations*: *CI *Confidence interval; *k*, number of studies; *n*, number of participants;^a^Random-effects model; ^b^Fixed-effects model; ^c^No pooled prevalence (only one study)

## Discussion

This systematic review and meta-analysis was the first to estimate the aggregate prevalence of symptoms during treatment in children with ALL. In children with ALL, 34 symptoms were identified. Symptoms remained highly prevalent in paediatric patients with ALL. The most prevalent symptoms were fatigue, lack of energy, dry mouth, lack of appetite, sweating, and feeling irritable, and these occurred in at least 60% of the children with ALL. We could not find additional meta-analysis studies on the incidence of symptoms during treatment in children with cancer; thus, comparisons could not be made. While compared to the systematic reviews of childhood cancer survivors, the prevalence rates of these symptoms were higher. Hong et al. reported a pooled prevalence of 39% for fatigue, 40% for dry mouth, 31% for lack of appetite, and 14% for sweating [32]. This result may be explained by the fact that some symptoms were resolved by treatment. It also suggested symptoms may persist for months to years after the completion of treatment. However, due to the patients analyzed in their study with different cancer diagnoses, we cannot draw definitive conclusions. Further research should focus on examining more homogeneous patient groups, which is a strength of the present review.

The current systematic review indicates that although children with ALL experienced multiple persistent adverse symptoms during therapy, they received disproportionate attention. Similar to previous research [32], the most studied physical symptoms were fatigue and sleep disturbance, and their biological mechanism [33–35], influence factor [19], and trajectories [36] had been explored. However, little is known about the biological mechanisms and management strategies of dry mouth, lack of appetite, and sweating, yet the prevalence of some of these symptoms was greater than the more commonly assessed symptoms. Similarly, psychological symptoms, including irritability (60.0%), worrying (42.5%), depression (27.6%), and anxiety (24.8%) have not received sufficient attention. The limited understanding of these symptoms may also partly explain the lack of effective management strategies to address them. Given the negative impact of symptom burden on the QOL, increased attention needs to be paid to these symptoms.

Since various tools with preselected lists of symptoms were used to collect data, symptoms not included in the lists were not measured. The symptoms identified in this study do not represent the complete symptom experience of children with ALL. Moreover, the MSAS 10–18 was the most commonly used scale to measure multiple symptoms. Although MSAS 10–18 has proven to be reliable and valid [37], it was originally developed for adults and might miss some essential dimensions of the symptom experiences in children. Therefore, concept elicitation interview [38] with ALL is recommended in future studies to enable researchers to develop an age-appropriate and accurately representative tool for symptom assessment.

### Limitations

This review had some limitations. First, only studies published in English or Chinese were included, causing a potential language bias. Second, inconsistencies existed in the assessment and reporting of symptoms across the included studies. Such inconsistencies and gaps led to variability between the studies. Computing the pooled mean frequency for several symptoms from disparate and incompatible data for conducting a meta-analysis is challenging. The evidence in this review is, therefore, weak, and the exact prevalence of symptoms in children with ALL during treatment remains to be determined.

### Clinical application

Results of this systematic review show that despite the development of new guidelines for symptom assessment and management [39, 40], the prevalence of symptoms is still high during ALL therapy. That might probably be because, in the past decades, most paediatric oncology research has focused on improving the cure rates, which led to the remarkable increase of the 5-year survival rate to 90% [3]. However, efforts to manage symptoms in children with cancer have not kept pace with new advances in the cure for childhood cancer. Nurses have a critical role in symptom assessment and management. Specific intervention is urgently needed to mitigate the symptoms in children with ALL and help them cope with the symptom burden.

## Conclusion

Our review provides a comprehensive overview of the existing literature with respect to the symptoms that children with ALL experience during treatment. Future research needs to explore interventions to improve the symptom burden, especially for symptoms that receive less attention at present, to minimize the symptom distress and improve the QoL of children.

### Supplementary Information

**Additional file 1: Supplemental Table S1.** Search strategy. **Supplemental Table S2.** Quality assessment of the included cross-sectional studies. **Supplemental Table S3.** Quality assessment of the included case-control study. **Supplemental Table S4.** Quality assessment of the included longitudinal study.

## Data Availability

The datasets used and/or analysed during the current study available from the corresponding author on reasonable request.
