# Supplementary material for: Prevalence of symptoms in children with acute lymphoblastic leukaemia: a systematic review and meta-analysis
Source: BMC Cancer. 2023 Nov 15;23:1113. doi: 10.1186/s12885-023-11581-z (PMC10648628; doi:10.1186/s12885-023-11581-z)
Supplement: Supplementary file 1 — Additional file 1: Supplemental Table S1. Search strategy. Supplemental Table S2. Quality assessment of the included cross-sectional studies. Supplemental Table S3. Quality assessment of the included case-control study. Supplemental Table S4. Quality assessment of the included longitudinal study. [file 12885_2023_11581_MOESM1_ESM.docx]

**Supplemental Table S1**

**Search strategy**

| Pubmed | #1 | "Signs and Symptoms"[Mesh] |
| --- | --- | --- |
|  | #2 | symptom*[Title/Abstract] OR problem*[Title/Abstract] OR consequenc*[Title/Abstract] OR side effect*[Title/Abstract] OR adverse effect*[Title/Abstract] OR secondary effect*[Title/Abstract] |
|  | #3 | "Leukemia"[Mesh] |
|  | #4 | leukem*[Title/Abstract] OR leukaem*[Title/Abstract] OR leucem*[Title/Abstract] |
|  | #5 | "Child"[Mesh] |
|  | #6 | "Child, Preschool"[Mesh] |
|  | #7 | "Adolescent"[Mesh] |
|  | #8 | "Pediatrics"[Mesh] |
|  | #9 | infan*[Title/Abstract] OR newborn*[Title/Abstract] OR perinat*[Title/Abstract] OR neonat*[Title/Abstract] OR baby[Title/Abstract] OR baby*[Title/Abstract] OR babies[Title/Abstract] OR toddler*[Title/Abstract] OR minors[Title/Abstract] OR minors*[Title/Abstract] OR boy[Title/Abstract] OR boys[Title/Abstract] OR boyfriend[Title/Abstract] OR boyhood[Title/Abstract] OR girl*[Title/Abstract] OR kid[Title/Abstract] OR kids[Title/Abstract] OR child[Title/Abstract] OR child*[Title/Abstract] OR children*[Title/Abstract] OR schoolchild*[Title/Abstract] OR schoolchild[Title/Abstract] OR school child[Title/Abstract] OR school child*[Title/Abstract] OR adolescen*[Title/Abstract] OR juvenil*[Title/Abstract] OR youth*[Title/Abstract] OR teen*[Title/Abstract] OR under* age*[Title/Abstract] OR pubescen*[Title/Abstract] OR pediatrics[Title/Abstract] OR pediatric*[Title/Abstract] OR paediatric*[Title/Abstract] OR peadiatric*[Title/Abstract] OR school[Title/Abstract] OR school*[Title/Abstract] OR prematur*[Title/Abstract] OR preterm*[Title/Abstract] |
|  | #10 | #1 OR #2 |
|  | #11 | #3 OR #4 |
|  | #12 | #5 OR #6 OR #7 OR #8 OR #9 |
|  | #13 | #10 AND #11 AND #12 |
|  | #14 | (#13) NOT (Review[Filter]) |
|  | #15 | (#14) NOT (Systematic Review[Filter]) |
|  | #16 | (#15) NOT (Meta-Analysis[Filter]) |
|  | #17 | (#16) NOT (Case Reports[Filter]) |
|  | #18 | (#17) NOT (Comment[Filter]) |
|  | #19 | (#17) NOT (Comment[Filter]) Filters: Journal Article, Humans, English, Child: birth-18 years |
| Web of Science | #1 | (infan* OR newborn* OR new‐born* OR perinat* OR neonat* OR baby OR baby* OR babies OR toddler* OR minors OR minors* OR boy OR boys OR boyfriend OR boyhood OR girl* OR kid OR kids OR child OR child* OR children* OR schoolchild* OR schoolchild OR school child OR school child* OR adolescen* OR juvenil* OR youth* OR teen* OR under*age* OR pubescen* OR pediatrics OR pediatric* OR paediatric* OR peadiatric* OR school OR school* OR prematur* OR preterm*) ti.ab |
|  | #2 | (leukem* or leukaem* or leucem*) ti.ab |
|  | #3 | (symptom* OR problem* OR consequenc* OR side effect* OR adverse effect* OR secondary effect*) ti.ab |
|  | #4 | #1 AND #2 AND #3 |
|  | Filter | Document type exclude (Review Articles or Meeting Abstracts or Proceedings Papers or Editorial Materials or Book Chapters or Letters or Corrections or Withdrawn Publication) |
|  | Filter | Languages Refine (English) |
| CINAHL | S1 | (MH "Signs and Symptoms+") |
|  | S2 | TI (symptom* OR problem* OR consequenc* OR side effect* OR adverse effect* OR secondary effect* ) OR AB ( symptom* OR problem* OR consequenc* OR side effect* OR adverse effect* OR secondary effect* ) |
|  | S3 | (MH "Child+") |
|  | S4 | (MH "Child, Preschool") |
|  | S5 | (MH "Adolescence+") |
|  | S6 | (MH "Pediatrics+") |
|  | S7 | TI ( infan* OR newborn* OR new‐born* OR perinat* OR neonat* OR baby OR baby* OR babies OR toddler* OR minors OR minors* OR boy OR boys OR boyfriend OR boyhood OR girl* OR kid OR kids OR child OR child* OR children* OR schoolchild* OR schoolchild OR school child OR school child* OR adolescen* OR juvenil* OR youth* OR teen* OR under*age* OR pubescen* OR pediatrics OR pediatric* OR paediatric* OR peadiatric* OR school OR school* OR prematur* OR preterm* ) OR AB ( infan* OR newborn* OR new‐born* OR perinat* OR neonat* OR baby OR baby* OR babies OR toddler* OR minors OR minors* OR boy OR boys OR boyfriend OR boyhood OR girl* OR kid OR kids OR child OR child* OR children* OR schoolchild* OR schoolchild OR school child OR school child* OR adolescen* OR juvenil* OR youth* OR teen* OR under*age* OR pubescen* OR pediatrics OR pediatric* OR paediatric* OR peadiatric* OR school OR school* OR prematur* OR preterm* ) |
|  | S8 | (MH "Leukemia+") |
|  | S9 | TI ( leukem* or leukaem* or leucem* ) OR AB ( leukem* or leukaem* or leucem* ) |
|  | S10 | S1 OR S2 |
|  | S11 | S3 OR S4 OR S5 OR S6 OR S7 |
|  | S12 | S8 OR S9 |
|  | S13 | S10AND S11 AND S12 |
| Embase | #1 | 'child'/exp |
|  | #2 | 'adolescent'/exp |
|  | #3 | 'pediatrics'/exp |
|  | #4 | infan*:ab,ti OR newborn*:ab,ti OR new‐ born*:ab,ti OR perinat*:ab,ti OR neonat*:ab,ti OR baby:ab,ti OR baby*:ab,ti OR babies:ab,ti OR toddler*:ab,ti OR minors:ab,ti OR minors*:ab,ti OR boy:ab,ti OR boys:ab,ti OR boyfriend:ab,ti OR boyhood:ab,ti OR girl*:ab,ti OR kid:ab,ti OR kids:ab,ti OR child:ab,ti OR child*:ab,ti OR children*:ab,ti OR schoolchild*:ab,ti OR schoolchild:ab,ti OR 'school child':ab,ti OR 'school child*':ab,ti OR adolescen*:ab,ti OR juvenil*:ab,ti OR youth*:ab,ti OR teen*:ab,ti OR under*age*:ab,ti OR pubescen*:ab,ti OR pediatrics:ab,ti OR pediatric*:ab,ti OR paediatric*:ab,ti OR peadiatric*:ab,ti OR school:ab,ti OR school*:ab,ti OR prematur*:ab,ti OR preterm*:ab,ti |
|  | #5 | #1 OR #2 OR #3 OR #4 |
|  | #6 | 'leukemia'/exp |
|  | #7 | leukem*:ab,ti OR leukaem*:ab,ti OR leucem*:ab,ti |
|  | #8 | #6 OR #7 |
|  | #9 | 'symptoms'/exp |
|  | #10 | symptom*:ab,ti OR problem*:ab,ti OR consequenc*:ab,ti OR 'side effect*':ab,ti OR 'adverse effect*':ab,ti OR 'secondary effect*':ab,ti |
|  | #11 | #9 OR #10 |
|  | #12 | #5AND #8AND #11 |
|  | #13 | #12AND 'childhood leukemia'/dm AND 'human'/de AND 'article'/it AND ([adolescent]/lim OR [child]/lim OR [infant]/lim OR [newborn]/lim OR [preschool]/lim OR (Wyatt et al.)/lim) |
| PsycInfo | 1 | child.mh. |
|  | 2 | Child, Preschool.mh. |
|  | 3 | Pediatrics.mh. |
|  | 4 | Adolescent.mh. |
|  | 5 | (infan* or newborn* or perinat* or neonat* or baby or baby* or babies or toddler* or minors or minors* or boy or boys or boyfriend or boyhood or girl* or kid or kids or child or child* or children* or schoolchild* or schoolchild or school child or school child* or adolescen* or juvenil* or youth* or teen* or under* age* or pubescen* or pediatrics or pediatric* or paediatric* or peadiatric* or school or school* or prematur* or preterm*).ab,ti. |
|  | 6 | leukemia.mh. |
|  | 7 | (leukem* or leukaem* or leucem*).ab,ti. |
|  | 8 | (Signs and Symptoms).mh. |
|  | 9 | (symptom* or problem* or consequenc* or side effect* or adverse effect* or secondary effect*).ab,ti. |
|  | 10 | 1 or 2 or 3 or 4 or 5 |
|  | 11 | 6 or 7 |
|  | 12 | 8 or 9 |
|  | 13 | 10 and 11 and 12 |
| CNKI | 1 | R:(leukemia) AND R:(children OR adolescent) AND R:(symptom) |
| China WanFang Database | 1 | R:(leukemia) AND R:(children OR adolescent) AND R:(symptom) |
| China Science and Technology Journal Database | 1 | R:(leukemia) AND R:(children OR adolescent) AND R:(symptom) |

**Supplemental Table S2 Quality assessment of the included cross-sectional studies**

| The Joanna Briggs Institute checklist question | | Zupanec et al | Bu et al. | Ren et al. | Daniel et al. | Fadhilah et al. | Loves et al. | Tomlinson et al. | Ma et al. | Hyslop et al. | Zhou et al. | Xi  et al., |
| --- | --- | --- | --- | --- | --- | --- | --- | --- | --- | --- | --- | --- |
|  |  | 2010 | 2015 | 2017 | 2018 | 2019 | 2019 | 2019 | 2019 | 2021 | 2021 | 2023 |
| 1 | Were the criteria for inclusion in the sample clearly defined? | Y | Y | Y | Y | Y | Y | Y | Y | Y | Y | Y |
| 2 | Were the study subjects and the setting described in detail? | Y | Y | Y | Y | Y | Y | Y | Y | Y | Y | Y |
| 3 | Was the exposure measured in a valid and reliable way? | Y | Y | Y | Y | Y | Y | Y | Y | Y | Y | Y |
| 4 | Were objective, standard criteria used for measurement of the condition? | Y | Y | Y | Y | Y | Y | Y | Y | Y | Y | Y |
| 5 | Were confounding factors identified? | Y | N | N | Y | Y | Y | N | Y | Y | Y | Y |
| 6 | Were strategies to deal with confounding factors stated? | Y | N | N | Y | Y | Y | N | Y | Y | Y | Y |
| 7 | Were the outcomes measured in a valid and reliable way? | Y | Y | Y | Y | Y | Y | Y | Y | Y | Y | Y |
| 8 | Was appropriate statistical analysis used? | Y | Y | Y | Y | Y | Y | Y | Y | Y | Y | Y |
| N, no; Y, yes. | | | | | | | | | | | | |

**Supplemental Table S3 Quality assessment of the included** **case-control study**

| The Joanna Briggs Institute checklist question | | McCarthy et al.  2016 |
| --- | --- | --- |
| 1 | Were the groups comparable other than the presence of disease in cases or the absence of disease in controls? | Y |
| 2 | Were cases and controls matched appropriately? | Y |
| 3 | Were the same criteria used for identification of cases and controls? | Y |
| 4 | Was exposure measured in a standard, valid and reliable way? | Y |
| 5 | Was exposure measured in the same way for cases and controls? | Y |
| 6 | Were confounding factors identified? | Y |
| 7 | Were strategies to deal with confounding factors stated? | Y |
| 8 | Were outcomes assessed in a standard, valid and reliable way for cases and controls? | Y |
| 9 | Was the exposure period of interest long enough to be meaningful? | Y |
| 10 | Was appropriate statistical analysis used? | Y |
| N, no; Y, yes. | | |

**Supplemental Table S4 Quality assessment of the included longitudinal study**

| The Joanna Briggs Institute checklist question | | Hockenberry et al. 2014 | Kunin-Batson et al. 2016 | Li et al. 2019 | Steur et al. 2020 | Irestorm et al. 2023 |
| --- | --- | --- | --- | --- | --- | --- |
| 1 | Were the criteria for inclusion in the sample clearly defined? | Y | Y | Y | Y | Y |
| 2 | Were the study subjects and the setting described in detail? | Y | Y | Y | Y | Y |
| 3 | Was the exposure measured in a valid and reliable way? | Y | Y | Y | Y | Y |
| 4 | Were confounding factors identified? | N | Y | N | Y | Y |
| 5 | Were strategies to deal with confounding factors stated? | N | Y | N | Y | Y |
| 6 | Were the outcomes measured in a valid and reliable way? | Y | Y | Y | Y | Y |
| 7 | Was the follow up time reported and sufficient to be long enough for outcomes to occur? | Y | Y | Y | Y | Y |
| 8 | Was follow up complete, and if not, were the reasons to loss to follow up described and explored? | Y | N | N | Y | Y |
| 9 | Were strategies to address incomplete follow up utilized? | N | Y | N | N | Y |
| 10 | Was appropriate statistical analysis used? | Y | Y | Y | Y | Y |
| N, no; Y, yes. | | | | | | |
